# Energy Consumption Forecasting Using Semantic-Based Genetic Programming with Local Search Optimizer

**DOI:** 10.1155/2015/971908

**Published:** 2015-05-28

**Authors:** Mauro Castelli, Leonardo Trujillo, Leonardo Vanneschi

**Affiliations:** ^1^NOVA IMS, Universidade Nova de Lisboa, 1070-312 Lisboa, Portugal; ^2^Tree-Lab Instituto Tecnológico de Tijuana, Mesa de Otay, 22500 Tijuana, BC, Mexico

## Abstract

Energy consumption forecasting (ECF) is an important policy issue in today's economies. An accurate ECF has great benefits for electric utilities and both negative and positive errors lead to increased operating costs. The paper proposes a semantic based genetic programming framework to address the ECF problem. In particular, we propose a system that finds (quasi-)perfect solutions with high probability and that generates models able to produce near optimal predictions also on unseen data. The framework blends a recently developed version of genetic programming that integrates semantic genetic operators with a local search method. The main idea in combining semantic genetic programming and a local searcher is to couple the exploration ability of the former with the exploitation ability of the latter. Experimental results confirm the suitability of the proposed method in predicting the energy consumption. In particular, the system produces a lower error with respect to the existing state-of-the art techniques used on the same dataset. More importantly, this case study has shown that including a local searcher in the geometric semantic genetic programming system can speed up the search process and can result in fitter models that are able to produce an accurate forecasting also on unseen data.

## 1. Introduction

As reported in [1, 2], energy consumption forecasting (ECF) is the task of predicting the electricity demand on different time scales, in minutes (very short-term), hours/days (short-term), months, and years (long-term). An accurate ECF has great benefits for electric utilities and both negative and positive errors lead to increased operating costs. Overestimating the energy demand leads to an unnecessary energy production or purchase and, on the contrary, underestimation causes unmet demand with a higher probability of failures and costly operations. With the recent trend of deregulation of electricity markets, ECF has gained even more importance. In particular, in a dynamic market environment, precise forecasting is the basis of electrical energy trade and spot price establishment for the system to gain the minimum electricity purchasing cost. At the same time, with the amount of data steadily growing, the problem is getting more and more complex. All these facts show the importance of having reliable predictive models that can be used for an accurate energy consumption forecasting [2]. Numerous contributions presenting computational intelligence (CI) based approaches for ECF have appeared in the last years [3]. Surveys can be found in [2, 4]. Among the different CI methods, particular importance was given to neural networks [5, 6], particle swarm optimization [7], support vector machines [8], simulated annealing [9], and genetic algorithms [10]. One of the outcomes of the European Energy Forecast conference [11] that took place in Brussels in February 2014 was the identification the following facts and open issues. (a) ECF will have a huge impact on economy in the near future. (b) ECF is a very difficult problem, since it is influenced by asynchronous and often unpredictable facts. (c) Several different geographical and time scales can be identified for ECF, which contribute to making the task even more complex. (d) The currently existing CI technologies are inadequate to take on this new challenge, making new computational methods much in demand. In particular, the following flaws of the current CI methods were identified.

1. Given the high complexity of ECF, the iterative process of stepwise improvement of solutions that characterizes many CI methods often gets stuck in local optima, stagnating the search for better solutions.
2. Several existing forecasting methods are not able to deal with nonlinearity and other difficulties in modelling of time series. Hence, the performance of these models on test data are not as good as the one achievable on the training data.

The objective of the work presented in this paper is to fill this gap by developing a ground-breaking Genetic Programming (GP) system that (i) finds (quasi-)perfect solutions with high probability (no error on training data) and (ii) generates models able to produce near optimal predictions also on unseen data (test instances). GP is one of the most successful existing CI methods. In the last years, it has obtained excellent results on a large number of complex real-life applications [12] and it has recently made an important breakthrough: the definition of geometric semantic operators (GSOs), new genetic operators that induce a unimodal error surface on any supervised learning problem (including forecasting). Eliminating local optima, GSOs have a stronger problem solving ability. So, they are an excellent first step for overcoming issue number (1) discussed above and developing appropriate forecasting models. However, much work has still to be done in order to use GSOs in a complex application like the ECF. In particular, GSOs converge to optimal solution(s) very slowly and this behaviour is an important limitation in all the applications characterized by the presence of a large amount of data. Hence, in this paper we propose the definition of a CI system that combines GSOs with a local search algorithm. The main idea in combining GSOs and a local searcher is to integrate the exploration ability of GSOs with the exploitation ability of the local searcher. In this way, we expect to achieve optimal solutions faster and to obtain a final model that does not overfit the training data. To analyze the appropriateness of the proposed computational method for ECF, the energy consumption in Italy has been used as a test case.

The paper is organized as follows.  Section 2 presents the variant of GP proposed in this study for addressing the ECF problem.  Section 3 describes the data that have been considered and reports experimental results, comparing the proposed approach to the standard GP algorithm and other state-of-the-art GP variants.  Section 4 concludes the paper, highlighting the main contributions of this work. In the final part of the paper, the appendix contains general introductions of basic concepts for nonexperts in the GP field.

## 2. Methodology

This section describes the components of the proposed computational intelligence system designed for the ECF problem. In particular, Section 2.1 describes the geometric semantic operators and their properties, while Section 2.2 presents the local search strategy that we used with the GSOs.

### 2.1. Geometric Semantic Operators

Despite the large number of human-competitive results achieved by GP [12], researchers still continue to investigate new methods for improving the power of GP as a problem solving method. In recent years, one of the emerging ideas is to include semantic awareness in the evolutionary process performed by GP. While several studies exist (a survey can be found in [13]), the definition of semantics is not unique and this concept is interpreted in different ways and under different perspectives [13]. In this work, we use the most common and widely accepted definition of semantics in the GP literature. Hence, the semantics of a program *T*_*i*_ is defined as the vector of outputs **s**_*i*_ = [*T*_*i*_(**x**_1_), *T*_*i*_(**x**_2_),…, *T*_*i*_(**x**_*n*_)] obtained after executing the program (or candidate solution) on a set of the training data *𝕋* = {**x**_1_, **x**_2_,…, **x**_*n*_}, such that **s**_*i*_ ∈ *ℝ* ^*n*^ [14].

In this section, we briefly describe the definition of the geometric semantic operators (GSOs) proposed by Moraglio and coauthors [14]. The objective of GSOs is to define modifications on the syntax of GP individuals that have a precise correspondence on their semantics. More in particular, the idea is to define transformations on the syntax of GP individuals that correspond to well known operators of genetic algorithms (GAs). In this way, GP could “inherit” the known properties of those GAs operators. Furthermore, contrarily to what typically happens in real-valued GAs or other heuristics, in the GP semantic space the target point is also known (it corresponds to the vector of expected output values in supervised learning) and the fitness of an individual is simply given by the distance between the point it represents in the semantic space and the target point (it corresponds to an error measure). The real-valued GA operators that we want to “map” into the GP semantic space are* geometric crossover* and* ball mutation*. In real-valued GAs, geometric crossover produces an offspring that stands in the segment that joins the parents. It was proven in [15] that in cases where the fitness is a direct function of the distance to the target (like the case we are interested in here) this offspring cannot have a worse fitness than the worst of its parents. Ball mutation consists in a random perturbation of the coordinates of an individual. It was shown in [14] that it induces a unimodal error surface for all the problems where fitness is a direct function of the distance to the target. The definitions of the operators that correspond to geometric crossover and ball mutation in the GP semantic space are as given in [14], respectively.

**Definition 1 (geometric semantic crossover (GSC)).**

Given two parent functions *T*_1_, *T*_2_ : *ℝ* ^*n*^ → *ℝ*, the geometric semantic crossover returns the real function *T*_*XO*_ = (*T*_1_ · *T*_*R*_)+((1 − *T*_*R*_) · *T*_2_), where *T*_*R*_ is a random real function whose output values range in the interval [0,1].

**Definition 2 (geometric semantic mutation (GSM)).**

Given a parent (as in [14], we abuse of the term “parent,” using it also to identify the solution that is transformed by a mutation operator) function *T* : *ℝ* ^*n*^ → *ℝ*, the geometric semantic mutation with mutation step ms returns the real function *T*_*M*_ = *T* + ms · (*T*_*R*1_ − *T*_*R*2_), where *T*_*R*1_ and *T*_*R*2_ are random real functions.

The interested reader is referred to [14] for a detailed discussion of these operators, including a justification of the fact that they correspond, respectively, to geometric crossover and ball mutation in the GP semantic space. Following [14], from now on GP that uses geometric semantic operators will be called geometric semantic GP (GSGP). As Moraglio et al. point out, geometric semantic operators create much larger offspring than their parents and the fast growth of the individuals in the population rapidly makes fitness evaluation unbearably slow, making the system unusable. Moreover, while this growth produces fitter solutions, it is responsible for creating models that are too specialized on training data, hence often generating overfitting. In [16], a possible workaround to the problem related to the slowness of the fitness evaluation process was proposed, consisting in an implementation of these operators that makes them not only usable in practice, but also very efficient. Basically, this implementation is based on the idea that, besides storing the initial trees, at every generation it is enough to maintain in memory, for each individual, its semantics and a reference to its parents. As shown in [16], the computational cost of evolving a population of *n* individuals for *g* generations is *O*(*ng*), while the cost of evaluating a new, unseen, instance is *O*(*g*). Hence, the system can be efficiently used to address problems characterized by a large amount of data. This is the implementation used in this work.

### 2.2. Local Search in Geometric Semantic Operators

In this work, we integrate a local search (LS) strategy within GSGP. In particular, we include a local searcher within the GSM mutation operator, since previous works have shown that GSGP achieves its best performance using only mutation [13]. In particular, the GSM with LS (GSM-LS) of a tree *T* generates an individual:

(1) $$\begin{array}{c} T_{M}=\alpha_{0}+\alpha_{1}\cdot T+\alpha_{2}\cdot \left(\;T_{R1}-T_{R2}\;\right), \end{array}$$

where *α*_*i*_ ∈ *ℝ*. Notice that *α*_2_ replaces the mutation step parameter ms used in the definition of GSM. Equation (1) defines a basic multivariate linear regression problem, which can be solved, for example, by Ordinary Least Square (OLS) regression. In this sense, after each mutation event, OLS is applied to the above expression to obtain the values of the model parameters (*α*_0_, *α*_1_, *α*_2_) that best fit the training fitness cases. We point out that, in some sense, this approach contrasts with previous work [17] that relied on a nonlinear local optimizer, since the linear assumption is mostly not satisfied by the expression evolved with standard GP and the corresponding parametrization. On the other hand, in this new approach it is simple to apply a linear regression optimizer, given that the GSM operator defines a linear expression in the parameter space. The idea of including a LS method is based on a very simple observation related to the properties of the geometric semantic operators: while these operators are effective in achieving good performance with respect to standard syntax-based operators, they require a lot of generations to converge to optimal solutions. Including a local search method, we expect to speed up the process and to obtain better solutions faster. Moreover, by speeding up the search process, it will be possible to limit the construction of overspecialized solutions that will eventually overfit the data.

## 3. Experimental Study

This section describes the data, the experimental settings, and the obtained results for the ECF problem.

### 3.1. Data Description

Historical energy consumption data and weather information in Italy in the years between 1999 and 2010 have been used to test the performance of the proposed system. TERNA S.p.A. (Rete Elettrica Nazionale) is an Italian electricity transmission system operator based in Rome, Italy. With 63,500 kilometres of power lines or around 98% of the Italian high-voltage power transmission grid, TERNA is the first independent electricity transmission grid operator in Europe and the sixth in world based on the size of its electrical grid. TERNA is the owner of the Italian transmission grid and responsible for energy transmission and dispatching. The aim of the forecasting task studied in this paper is to predict the energy consumption at day *t*, providing information until day *t* − 1 (one-day ahead forecasting) using the past samples of the load and weather information. Data include temperatures, pressure values, wind speed, and other weather related information. Data from 1999 to 2006 have been used during the training phase, while the remaining available data (i.e., from 2006 to 2010) have been used to validate the model on unseen data and hence to assess the quality of the forecasting. The same dataset has been used in [1], where the standard GSGP system has been used for the same task and where GSGP was able to outperform state-of-the-art machine learning techniques in the ECF problem. Hence, in the presentation of the results, it will be interesting to assess whether or not the inclusion of a local searcher optimizer is able to produce a competitive advantage with respect to the simple use of GSGP.

### 3.2. Experimental Settings

Four different GP systems were compared: standard GP (STGP) that uses the standard syntax-based genetic operators also considered in [1], GSGP that only uses the GSM operator; HYBRID that uses the GSM operator and the proposed GSM-LS operator, LSGP that only uses the GSM-LS operator at each generation of the evolutionary search process.

Regarding the four GP systems, all the runs used populations of 200 individuals allowed to evolve for 50 generations. Tree initialization was performed with the Ramped Half-and-Half method [18] with a maximum initial depth equal to 6. The function set contained the arithmetic operators, including protected division as in [18]. The terminal set contained 45 variables, each one corresponding to a different feature in the dataset. Mutation has been used with probability 1, while in STGP we used a mutation rate of 0.4 and a crossover rate of 0.6. The use of different settings for STGP is motivated by the fact that STGP with only mutation performs poorly on this problem. Hence, we decided to consider the settings that are able to produce the best performance for each of the studied systems. Survival from one generation to the other was always guaranteed to the best individual of the population (elitism). For GSM, a random mutation step has been considered in each mutation event, as suggested in [13]. Regarding the HYBRID system, GSM-LS has been used in the first 20 generations, while in the remaining generations we considered the standard GSM operator. We decided to limit the number of generations where the local search has been used in order to assess whether the HYBRID system produces “similar” results with respect to GSGP or LSGP.

For all the considered techniques we studied the obtained performance over two different measures of error. In particular, these two measures are the mean absolute error (MAE) and the mean square error (MSE). The definition of these error measures is as follows:

(2) MAE1N∑i∈Qti−yi,MSE=1N∑i∈Qti−yi2,

where *y*_*i*_ = *T*(**x**_*i*_) is the output of the GP individual *T* on the data sample **x**_*i*_ and *t*_*i*_ is the target value corresponding to **x**_*i*_. *N* denotes the number of samples in the training or testing subset, and *Q* contains the indices of that set.

In the next section, the obtained experimental results are reported using curves of the median error on the training and test set. In particular, at each generation the best individual in the population (i.e., the one that has the smaller training error) has been chosen and the value of its error on the training and test sets has been stored. The reported curves finally contain the median of all these values collected at each generation. The median was preferred over the mean in the reported plots because of its higher robustness to outliers. The results discussed in the next section have been obtained using the GSGP implementation freely available at http://gsgp.sourceforge.net and documented in [16].

### 3.3. Experimental Results

**Figure 1:**
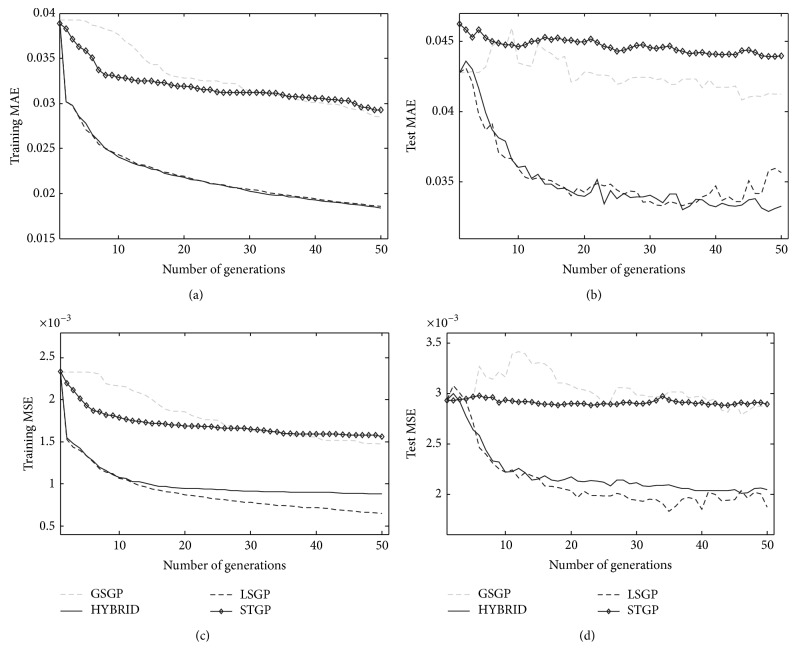
Training (plots (a) and (c)) and test (plots (b) and (d)) error for MAE (plots (a) and (b)) and MSE (plots (c) and (d)). The plots show the median over 30 independent runs.

Figure 1 reports training and test error (MAE and MSE) for the considered GP systems against generations. For all the considered GP systems 30 runs have been performed. These figures clearly show that LSGP outperforms GSGP and STGP on both training and test sets, for both the considered error measures. In particular, it is possible to note the fast convergence of the proposed system as well as the fact that the final model does not overfit the training data. A further corroboration about the suitability of combining GSGP with a local search optimizer is given by the performance of the HYBRID system. As it is possible to see from the figures, its performance is similar to the one achieved with LSGP, on both training and test data.

To analyze the statistical significance of these results, a set of tests has been performed on the median errors. In particular, we want to assess whether the final results (generation 50), produced by the considered GP systems, have a statistically significant difference. As a first step, the Shapiro Wilk test (with *α* = 0.1) has shown that the data are not normally distributed and hence a rank-based statistic has been used. Then, the Friedman test has been used. The null hypotheses for the comparison across repeated measures are that the distributions are the same across repeated measures. The alternative hypotheses is that distributions across repeated measures are different. Also in this test a value of *α* = 0.1 has been used and the Holm post hoc procedure has been considered. The *p* values obtained are reported in Table 1. According to the *p* values, we can clearly state that LSGP produces solutions that are significantly better (i.e., with lower error) than GSGP and STGP on both training and test data and for both the considered error measures. Also, the HYBRID method produces statistically better results with respect to GSGP and STGP. When LSGP is compared against HYBRID, it produces comparable results. The only difference that is statistically significant is the one related to the training fitness when the MSE is used as error measure. This last result is quite interesting, because it suggests that it is possible to achieve better results (with respect to the ones achieved with GSGP), by using the proposed GSM-LS operator only in the initial phase of the evolutionary search process, hence saving the computational time needed to run the local searcher in the subsequent generations.

To conclude the analysis of the experimental results, Table 2 reports the median (calculated over 30 runs) execution time of the considered systems as well as the standard deviation of the execution time. This comparison allows GP practitioners to compare the solution quality gain versus the required execution time. All the data are expressed in seconds. As it is possible to note, STGP is the technique that requires the largest amount of time to complete a run. This is an expected result that has been deeply discussed in [19]. Regarding the semantic-based systems, it seems that, while the inclusion of the local search method significantly improves the performance of GP, it has a negligible impact on the execution time. To strengthen this result, we performed a set of runs considering 2000 generations for both the GSGP and LSGP systems. In this case the median execution time was 89 seconds for GSGP and 93 seconds for LSGP. Hence, we conclude that LSGP has a competitive ratio between solution quality and required execution time with respect to the GSGP system, at least for the studied problem.

## 4. Conclusions

Electricity consumption forecasting (ECF) is important for the power industry, especially in the context of the ongoing deregulation of the electricity market. Proper demand forecasts help the market participants to maximize their profits and/or reduce their possible losses by preparing an appropriate bidding strategy. In this study, the ECF problem has been considered and in order to address it a computational intelligence technique has been proposed. The proposed system is based on a variant of the Genetic Programming (GP) algorithm. In particular, the GP system uses particular genetic operators that, differently from the standard genetic operators used in GP, work on the semantics of the solutions. While the use of semantic methods in GP has been successfully investigated and applied, several important problems that do not allow us to efficiently use these methods are still open. In particular, the GP system that uses the semantic operators (GSGP) requires a huge amount of generations to converge towards optimal solutions and, moreover, by producing a (quasi) optimal fitting of the training data it often generates solutions that are not able to generalize well over unseen instances. Under this light, the contribution of this work consists in integrating the GSGP framework with a local search optimizer. The use of a local searcher is motivated by the improvement of convergence speed of GSGP towards good quality solutions. Thus, by combining the exploration ability of GSGP with the exploitation ability of a local search method we expect to find good quality solutions in a small number of generations, hence avoiding the excessive specialization of a model on the training instances and, consequently, overfitting.

To validate the proposed system, called LSGP, an extensive experimental analysis has been performed, considering electricity consumption data that cover the period 1999–2010 in the Italian territory. We tested three semantic-based GP systems (GSGP, HYBRID, and LSGP) and a standard, syntax-based, GP system (STGP). GSGP is the GP system that uses the geometric semantic mutation defined in [14]; LSGP uses the GSM-LS mutation introduced in this work, while the hybrid system uses the GSM-LS operator in the initial generations and then it uses the GSM operator in the rest of the run. The reported results have shown that LSGP is able to produce results that are statistically better than the ones produced by GSGP that, as reported in [1], represents one of the state-of-the-art methods for addressing the ECF problem. In particular, LSGP is able to reduce the forecasting error with respect to GSGP and STGP, thus generating more accurate and reliable predictive models, without overfitting the training data. Moreover, LSGP (like the HYBRID system) outperforms GSGP and STGP also on test instances. Finally, the HYBRID system produces similar performance with respect to LSGP. Hence, also using the GSM-LS operator only at the beginning of the search process results in a more accurate and reliable model with respect to the one obtained with GSGP.

To summarize, the paper provides two contributions: from the point of view of the energy consumption forecasting, a system that is able to outperform the existing state-of-the-art technique has been defined; from the machine learning perspective, this case study has shown that including a local searcher in the geometric semantic GP system can speed up the convergence of the search process, without a corresponding overfitting of training data. We hope that this contribution will pave the way for further research on these topics.

## Figures and Tables

**Figure 2:**
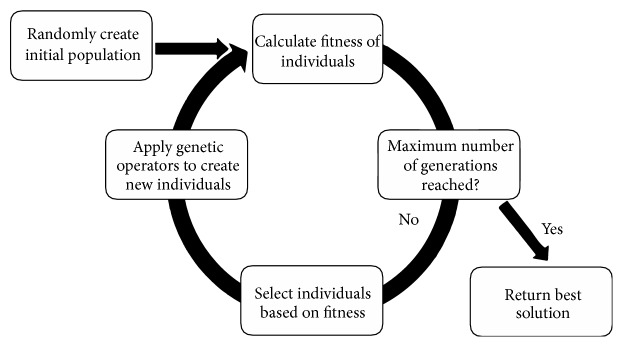
The GP algorithm.

**Table 1:** *p* values obtained from the statistical validation procedure.

	Training			Test		
	LSGP	STGP	HYBRID	LSGP	STGP	HYBRID
MAE						
GSGP	3.02*E* − 11	9.8*E* − 01	3.02*E* − 11	5.19*E* − 03	3.3*E* − 02	7.66*E* − 05
HYBRID	1.6*E* − 01	3.02*E* − 11	—	1.7*E* − 01	2.0*E* − 03	—
STGP	3.02*E* − 11	—	—	2.0*E* − 03	—	—

MSE						
GSGP	3.02*E* − 11	6.6*E* − 01	3.02*E* − 11	7.70*E* − 04	7.8*E* − 01	3.81*E* − 07
HYBRID	3.02*E* − 11	3.02*E* − 11	—	4.6*E* − 01	9.51*E* − 06	—
STGP	3.02*E* − 11	—	—	7.70*E* − 04	—	—

**Table 2:** Execution time (seconds) of the considered GP systems. Median and standard deviation calculated over 30 runs.

	MAE				MSE			
	GSGP	HYBRID	LSGP	STGP	GSGP	HYBRID	LSGP	STGP
Execution time	2.22	2.32	2.35	3.94	2.19	2.2	2.36	4.2
Standard dev.	0.12	0.11	0.12	0.83	0.14	0.13	0.13	0.78

## Appendix

### Genetic Programming

Genetic Programming (GP) is one of the techniques that belong to the computational intelligence research area called evolutionary computation. GP consists in the automated learning of computer programs by means of a process inspired by biological evolution [18]. Generation by generation, GP stochastically transforms populations of programs into new, hopefully improved, populations of programs. The quality of a solution is expressed by using an objective function (also called fitness function). The search process of GP is graphically depicted in Figure 2.

Hence, the recipe for solving a problem with GP is as follows.

- Choose a representation space in which candidate solutions can be specified. This consists of deciding on the primitives of the programming language that will be used to construct programs. A program is built up from a terminal set (the variables in the problem and, optionally, a set of constant values) and a function set (the primitive operators).
- Design the fitness criteria for evaluating the quality of a solution. This involves the execution of a candidate solution on a suite of test cases, reminiscent of the process of black-box testing. In case of supervised learning, a distance-based function is employed to quantify the divergence of a candidate's behavior from the desired one.
- Design a parent selection and replacement policy. Central to every evolutionary algorithm is the concept of fitness-driven selection in order to exert an evolutionary pressure towards promising areas of the program space. The replacement policy determines the way in which newly created offspring programs replace their parents in the population.
- Design a variation mechanism for generating offspring from a parent or a set of parents. Standard GP uses two main variation operators: crossover and mutation. Crossover recombines parts of the structure of two individuals, whereas mutation stochastically alters a portion of the structure of an individual.
- After a random initialization of a population of computer programs, an iterative application of selection-variation-replacement is employed to improve the programs quality in a stepwise refinement way.

In order to transform a population, GP uses genetic operators. Considering the common tree representation of GP individuals, the standard genetic operators (crossover and mutation) act on the structure of the trees that represent the candidate solutions. In other terms, standard genetic operators act on the syntax of the programs. In this paper, we used genetic operators that, differently from the standard ones, are able to act at the semantic level. The definition of semantics used in this work is the one also proposed in [14] and it is discussed in Section 2.1.

However, to understand the differences between the genetic operators used in this work and the ones used in the standard GP algorithm, the latter are briefly described. The standard crossover operator is traditionally used to combine the genetic material of two parents by swapping a part of one parent with a part of the other. More in detail, after choosing two individuals based on their fitness, the crossover operator performs the following operations: (1) it selects a random subtree in each parent and (2) swaps the selected subtrees between the two parents (the resulting individuals are the children). The mutation operator introduces random changes in the structures of the individuals in the population. The most well-known mutation operator, called subtree mutation, works as follows: (1) it randomly selects a point in a tree, (2) it removes whatever is currently at the selected point and whatever is below the selected point, and (3) it inserts a randomly generated tree at that point. This operation is controlled by a parameter that specifies the maximum size (usually measured in terms of tree depth) for the newly created subtree that is to be inserted.

*Symbolic Regression with Genetic Programming*. In symbolic regression, the goal is to search for the symbolic expression *T* ^*O*^ : *ℝ* ^*p*^ → *ℝ* that best fits a particular training set *𝕋* = {(**x**_1_, *t*_1_),…, (**x**_*n*_, *t*_*n*_)} of *n* input/output pairs with **x**_*i*_ ∈ *ℝ* ^*p*^ and *t*_*i*_ ∈ *ℝ*. The general symbolic regression problem can then be defined as

(A.1) TO,θO⟵arg minT∈G;θ∈Rm⁡fTxi,θ,tiwith  i=1,…,p,

where *𝔾* is the solution or syntactic space defined by the primitive set *ℙ* (functions and terminals), *f* is the fitness function based on the distance or error between a program's output *T*(**x**_*i*_, ***θ***) and the expected, or target, output *t*_*i*_, and ***θ*** is a particular parametrization of the symbolic expression *T*, assuming *m* real-valued parameters. In standard GP, parameter optimization is usually not performed explicitly, since GP search operators only focus on syntax. Therefore, the parameters are only implicitly considered. However, recent works have begun to address this issue, such as in [17], where a nonlinear numerical optimizer is used to tune the parametrization of the evolved programs, achieving substantial improvements in terms of convergence speed and solution quality. Let us consider the following hypothetical example to grasp the importance of such a process. Imagine a GP individual with a syntax *T*(*x*) = *x* + sin(*x*) and the following parametrization: ***θ*** = (*α*_1_, *α*_2_, *α*_3_), with *T*(*x*) = *α*_1_ *x* + *α*_2_sin(*α*_3_ *x*). In a traditional GP, these parameters are usually set to 1, which does not necessarily lead to the best possible performance for this particular syntax. Indeed, if the optimal solution is, for instance, *T* ^*O*^(*x*) = 3.3*x* + 1.003sin(0.0001*x*), then individual *T* might be easily discarded by the search, even though it has a quite similar shape to *T* ^*O*^. On the other hand, a local search process that performs a numerical optimization of these implicit parameters might be able to tune them and produce a substantial improvement in programs performance, potentially improving the fitness assigned to the above syntax. This is the view taken in this work, and while previous works have included parameter optimization for a standard GP search [17], this work applies it to GP that uses the new semantic-based genetic operators.
